# Clinical application of combined anticoagulation therapy in anti-MDA5 antibody-positive associated interstitial lung disease-a retrospective study

**DOI:** 10.3389/fphar.2025.1662306

**Published:** 2025-12-01

**Authors:** Ting Zhang, Yuanyuan Song, Hua Ma, Lina Cao, Li Lu, Hai Tang, Dongmei Zhou

**Affiliations:** 1 Department of Rheumatology and Immunology, Xuzhou Medical University Affiliated Hospital, Xuzhou, Jiangsu, China; 2 Department of Neurology, Xuzhou Medical University Affiliated Hospital, Xuzhou, Jiangsu, China

**Keywords:** anti-MDA5 antibody, interstitial lung disease, combined anticoagulation, MAD5, pulmonary function

## Abstract

**Objective:**

To explore the clinical efficacy of combined anticoagulation therapy in patients with anti-Melanoma Differentiation-Associated protein 5 (MDA5) antibody-positive associated interstitial lung disease.

**Methods:**

A retrospective cohort study was conducted, which included 100 patients diagnosed with anti-MDA5 antibody-positive interstitial lung disease at our hospital between January 2022 and December 2024. Based on the treatment approach, the patients were divided into two groups: the combined anticoagulation group (receiving conventional therapy plus sequential low-molecular-weight heparin followed by rivaroxaban) and the control group (receiving conventional therapy only). Comparisons were made between the two groups regarding pulmonary function parameters (FVC%, DLCO%, PaO_2_), inflammatory markers (CRP, ESR, LDH, CK), as well as the incidence of complications and acute exacerbations. Furthermore, logistic regression analysis was employed to identify independent risk factors for acute exacerbations.

**Results:**

At 4-week treatment and 3-month follow-up, the combined anticoagulation group had superior Forced Vital Capacity (FVC)%, Diffusing Capacity of the Lung for Carbon Monoxide (DLCO)%, and Arterial Partial Pressure of Oxygen (PaO_2_) levels compared to the control group (*P* < 0.05). Meanwhile, C-Reactive Protein (CRP), Lactate Dehydrogenase (LDH), and Creatine Kinase (CK) levels decreased significantly. Also, the incidence of complications and acute exacerbation was lower in the combined group (*P* < 0.05). Logistic regression showed decreased PaO_2_, elevated CRP, LDH, and CK, fungal infection, abnormal liver/kidney function, and non-combined anticoagulation as independent acute-exacerbation risk factors.

**Conclusion:**

Combined anticoagulation therapy improves pulmonary function, reduces inflammation, and lowers complication and exacerbation risks in anti-MDA5 antibody-positive interstitial lung disease patients. With notable clinical value, it offers a new treatment strategy for this refractory disease.

## Introduction

Anti-melanoma differentiation-associated gene 5 antibody (anti- Melanoma Differentiation-Associated protein 5 (MDA5) antibody)-positive myositis complicated with interstitial lung disease (referred to as anti-MDA5 antibody-positive interstitial lung disease) is a rapidly progressive inflammatory lung disease subtype with a poor prognosis. In recent years, its incidence has been increasing, particularly in the Asian population (Juan Zong et al., 2024). Studies have shown that anti-MDA5 antibody-related lung disease involves autoimmune-induced inflammatory responses, as well as pathological changes such as microvascular damage, abnormal vascular endothelial function, and alveolar capillary microthrombosis. Therefore, anticoagulation therapy may serve as a novel intervention method (Yan Xu and Lu, 2024).

Given this background, this study retrospectively analyzed the clinical data of patients diagnosed with anti-MDA5 antibody-positive associated interstitial lung disease in our hospital from January 2022 to December 2024. The aim was to explore the feasibility and efficacy of combined anticoagulation therapy as an adjunct to conventional treatment, thereby providing a basis for clinical decision-making.

## Materials and methods

### Participants

This was a retrospective controlled study involving 100 patients with anti-MDA5 antibody-positive interstitial lung disease admitted to the Department of Rheumatology and Immunology and the Department of Respiratory and Critical Care Medicine of our hospital from January 2022 to December 2024. Based on the treatment regimen, the patients were divided into two groups: the observation group (combined anticoagulation therapy, n = 52) and the control group (conventional treatment, n = 48). The study flowchart detailing patient selection and grouping is provided in Supplementary Figure S1.

In the observation group, there were 21 males and 31 females, with an average age of 51.77 ± 9.85 years (range: 32–74 years) and an average disease duration of 3.23 ± 1.12 months (range: 1.0–6.5 months). In the control group, there were 20 males and 28 females, with an average age of 52.91 ± 10.42 years (range: 33–72 years) and an average disease duration of 3.34 ± 1.17 months (range: 1.2–9.0 months). No significant differences were observed in gender, age, or disease duration between the two groups (*P* > 0.05), indicating comparability.

This study was approved by the Medical Ethics Committee of Xuzhou Medical University Affiliated Hospital (ethical approval number: YXYY-2021-KY023). All patients provided informed consent, and the study was performed in strict accordance with the Declaration of Helsinki, Ethical Principles for Medical Research Involving Human Subjects.

### Inclusion criteria

Patients were required to have a confirmed positive anti-MDA5 antibody status by immunological testing, along with a diagnosis of dermatomyositis. This diagnosis was based on the *2022 Chinese Consensus on the Diagnosis and Treatment of Anti-MDA5 Antibody-Positive Dermatomyositis* (Long, 2024).

The presence of interstitial lung disease (ILD) was defined by chest HRCT showing new-onset or progressive interstitial lung lesions. The specific imaging features included ground-glass opacities, reticular opacities, traction bronchiectasis, or honeycombing, in accordance with the imaging criteria outlined in the *2011 ATS/ERS/JRS/ALAT guideline for the diagnosis and management of IPF* (Supplementary Table S1) (Raghu et al., 2011).

Other secondary causes of lung lesions were rigorously excluded.

### Exclusion criteria

1. Patients diagnosed with other connective tissue diseases.
2. Patients with infectious lung diseases such as active tuberculosis, HIV, or fungal pneumonia.
3. Patients with a history of hemorrhagic diseases or abnormal coagulation function (PT prolonged > 3s, elevated D-dimer).
4. Patients with chronic liver or kidney failure, heart failure, or malignant tumors.
5. Patients with active pulmonary or systemic infections at baseline.

### Research method

This was a single-center retrospective-controlled analysis. All patients received a baseline treatment regimen of glucocorticoids combined with immunosuppressants:

Glucocorticoids: Initial dose of methylprednisolone 1.0 mg/kg·d via intravenous injection, gradually tapered to an oral maintenance dose (approximately 0.5 mg/kg·d) after stabilization.

Immunosuppressants: Intravenous cyclophosphamide (0.4 g/m^2^ every 2–3 weeks, for a total of 4–6 doses).

The observation group received additional anticoagulation therapy:

1. Low-molecular-weight heparin sodium (enoxaparin) 4,000 IU/d subcutaneously for 24 consecutive weeks.
2. Some patients were switched to oral rivaroxaban (10 mg/d) after discharge and maintained the treatment for at least 3 months.

During anticoagulation, D-dimer, Prothrombin Time (PT), Activated Partial Thromboplastin Time (APTT), Fibrinogen (FIB), platelet count, and liver/kidney function were monitored regularly. Treatment was adjusted or discontinued in cases of bleeding risk or adverse reactions.

The initial methylprednisolone dose (1.0 mg/kg·d) was maintained for 2 weeks, then tapered by 20% every 2 weeks based on clinical response, reaching a maintenance dose of 0.5 mg/kg·d by week 8.

### Observation indicators

#### Pulmonary function assessment

The main indicators include:

Forced vital capacity in the first second as a percentage of the predicted value (Forced Vital Capacity (FVC)%); Carbon monoxide diffusing capacity as a percentage of the predicted value (Diffusing Capacity of the Lung for Carbon Monoxide (DLCO)%); Arterial partial pressure of oxygen (Arterial Partial Pressure of Oxygen (PaO_2_)). The above indicators were measured before treatment, at 4 weeks of treatment, and at 3 months of follow-up.

#### Inflammatory and immune markers

The detection indicators include: C-reactive protein (CRP); erythrocyte sedimentation rate (ESR); lactate dehydrogenase (LDH) and creatine kinase (CK).

#### Assessment of the incidence of complications

The following complications were observed: bleeding events; pulmonary infections; fungal infections; liver function damage and renal function abnormalities.

#### Assessment of the incidence of acute exacerbation

Acute exacerbation was defined as meeting the following three criteria:

Obvious aggravation of dyspnea in a short period (<30 days); A decrease in PaO_2_ > 10 mmHg or the need for additional oxygen inhalation; Chest HRCT showed new-onset extensive ground-glass opacities or consolidation shadows.

### Statistical analysis

SPSS 24.0 was used for analysis. Measurement data were expressed as mean ± standard deviation (x̄ ± s) and compared using independent t-tests. Count data were expressed as frequency (n, %) and compared using chi-square tests. Binary logistic regression identified independent risk factors for acute exacerbation. *P* < 0.05 was considered statistically significant. We performed a *post hoc* power analysis using G*Power 3.1 software, which indicated that our sample size provided 85% power to detect the observed differences in FVC% at α = 0.05. To control for the potential confounding effect of infections on inflammatory markers, a prespecified subgroup analysis was performed on patients who did not develop any infections during the study period.

## Results

### Changes in pulmonary function

At 4 weeks, the combined anticoagulation group showed superior FVC% (68.34 ± 7.95 vs. 63.17 ± 8.16), DLCO% (63.87 ± 8.33 vs. 58.04 ± 9.11), and PaO_2_ (80.12 ± 6.73 vs. 75.69 ± 6.82 mmHg) compared to the control group (*P* < 0.01). At 3 months, FVC% increased to 70.41 ± 8.02 (14.4% improvement from baseline), and DLCO% reached 66.45 ± 7.85, demonstrating significant improvement in pulmonary function (Table 1).

**TABLE 1:** Comparison of pulmonary function changes between the combined anticoagulation group and the control group.

Indicator	Timepoint	Anticoagulation group (n = 50)	Control group (n = 50)	t	P
FVC%	Before treatment	61.52 ± 8.76	60.93 ± 8.41	0.342	>0.05
	Week 4	68.34 ± 7.95	63.17 ± 8.16	2.922	<0.01
	Month 3	70.41 ± 8.02	64.28 ± 7.93	3.821	<0.001
DLCO%	Before treatment	57.43 ± 9.21	56.22 ± 8.97	0.586	>0.05
	Week 4	63.87 ± 8.33	58.04 ± 9.11	3.144	<0.01
	Month 3	66.45 ± 7.85	59.38 ± 8.34	3.948	<0.001
PaO_2_	Before treatment	72.65 ± 7.89	71.38 ± 7.55	0.821	>0.05
	Week 4	80.12 ± 6.73	75.69 ± 6.82	3.261	<0.01
	Month 3	83.29 ± 6.44	77.83 ± 6.51	4.174	<0.001

### Changes in inflammatory and immune indicators

No baseline differences were observed (P > 0.05). At 4 weeks and 3 months, the combined anticoagulation group showed greater reductions in CRP, ESR, LDH, and CK (*P* < 0.05, Table 2).

**TABLE 2:** Evaluation of inflammatory immune response indicators of the two groups of patients.

Indicator	Timepoint	Anticoagulation group (n = 50)	Control group (n = 50)	t	P
CRP (mg/L)	Before treatment	29.65 ± 8.42	28.93 ± 7.88	0.45	>0.05
	Week 4	16.24 ± 5.31	21.17 ± 6.02	4.28	<0.001
	Month 3	12.81 ± 4.76	18.95 ± 5.12	5.67	<0.001
ESR (mm/h)	Before treatment	43.29 ± 10.57	41.87 ± 11.04	0.68	>0.05
	Week 4	28.13 ± 7.91	35.48 ± 8.03	4.76	<0.001
	Month 3	21.34 ± 6.52	30.69 ± 6.83	6.82	<0.001
LDH (U/L)	Before treatment	327.51 ± 54.83	329.37 ± 56.10	0.16	>0.05
	Week 4	261.45 ± 42.16	289.13 ± 44.21	3.24	<0.01
	Month 3	239.02 ± 40.39	278.56 ± 41.75	4.93	<0.001

There was no significant difference in baseline serum ferritin between the two groups.

At the 3-month follow-up, the combined anticoagulation group exhibited a significantly greater reduction in serum ferritin levels compared to the control group.

### Subgroup analysis excluding patients with infections

To ascertain that the reduction in inflammatory markers was independent of concurrent infections, we analyzed data from the subgroup of patients who did not develop infections (n = 77). In this infection-free cohort, the reductions in CRP and ESR at both the 4-week and 3-month timepoints remained significantly greater in the combined anticoagulation group compared to the control group (all P < 0.05). These results suggest that the anti-inflammatory effect of the anticoagulation therapy is not confounded by intercurrent infections.

### Incidence of complications

The total complication rate was lower in the combined anticoagulation group (23.0% vs. 74.0%). Specifically, pulmonary infections (8.0% vs. 22.0%), fungal infections (4.0% vs. 16.0%), and liver/kidney dysfunction were reduced (*P* < 0.05). Although the incidence of bleeding events was low and not statistically different between groups (6.0% vs. 4.0%, Table 3), it is noteworthy that the three bleeding events in the anticoagulation group (all mild gingival bleeding or epistaxis) resolved completely after permanent discontinuation of the anticoagulant, with no recurrence. No major bleeding events occurred.

**TABLE 3:** Evaluation of the incidence of concurrent disease in the two groups of patients.

Complication type	Anticoagulation group (n = 50)	Control group (n = 50)	*χ* ^2^	P
Hemorrhagic event	3 (6.0%)	2 (4.0%)	0.222	>0.05
Pulmonary infection	4 (8.0%)	11 (22.0%)	4.051	<0.05
Fungal infection	2 (4.0%)	8 (16.0%)	4.003	<0.05
Liver function injury	3 (6.0%)	9 (18.0%)	3.277	<0.05
Renal dysfunction	2 (4.0%)	7 (14.0%)	3.113	<0.05

### Incidence of acute exacerbation

The incidence of acute exacerbation was lower in the combined anticoagulation group (10.0% vs. 28.0%, *P* < 0.05). Logistic regression showed non-anticoagulation therapy had an Odds Ratio (OR) of 4.928 (95% Confidence Interval (CI): 1.553–15.645), higher than other risk factors (Table 4).

**TABLE 4:** Comparison of the incidence of acute exacerbation between the two groups of patients (number of cases/%).

Group	Number of patients (n)	Acute exacerbation [n (%)]
Anticoagulation group	50	5 (10.0%)
Control group	50	14 (28.0%)
*χ* ^2^		5.003
P		<0.05

### Logistic regression analysis

To further clarify the influencing factors of acute exacerbation in patients with anti-MDA5 antibody-positive interstitial lung disease, this study used “whether acute exacerbation occurred” as the dependent variable (exacerbation = 1, no exacerbation = 0), and selected representative variables from four dimensions including pulmonary function, inflammatory and immune status, complications, and treatment methods as independent variables for binary Logistic regression analysis. The results showed that decreased PaO_2_, increased CRP, increased LDH, increased CK, fungal infection, liver function damage, abnormal kidney function, and non-combined anticoagulation therapy were all independent risk factors for acute exacerbation (*P* < 0.05, Table 5).

**TABLE 5:** Multivariable logistic regression analysis of independent risk factors for acute exacerbation.

Variable	β	SE	Wald *χ* ^2^	OR (95% CI)	P
Non-combined anticoagulation therapy	1.72	0.61	7.98	5.58 (1.69–18.41)	0.005
PaO_2_ < 80 mmHg	1.35	0.58	5.41	3.86 (1.24–12.02)	0.020
CRP ≥ 20 mg/L	1.48	0.60	6.08	4.39 (1.35–14.27)	0.014
Fungal infection	1.60	0.65	6.06	4.95 (1.39–17.66)	0.014

The model was constructed with 4 variables based on 19 acute exacerbation events.

Abbreviations: CI, confidence interval; OR, odds ratio; Ref, Reference group.

## Discussion

Interstitial lung disease (ILD) is a heterogeneous lung disorder characterized by diffuse inflammation and/or fibrosis of the alveolar walls. Data from a multicenter clinical study in China demonstrated that up to 40% of patients with anti-MDA5 antibody-positive dermatomyositis developed rapidly progressive ILD (RP-ILD), with a 1-year mortality rate exceeding 50% (Jade et al., 2025; Yashan Zhou and Duan, 2024). The pathogenesis involves multiple factors, including abnormal immune regulation, vascular endothelial damage, and alveolar microthrombosis, suggesting limitations in traditional glucocorticoid and immunosuppressant regimens. Based on these findings, this study investigated the clinical value of adding anticoagulation therapy to conventional treatment. By systematically evaluating key indicators such as pulmonary function, inflammatory markers, and acute exacerbation incidence, we aimed to develop a novel therapeutic strategy for this severe disease subtype.

The results showed that the combined anticoagulation group exhibited significant improvements in pulmonary function parameters (FVC%, DLCO%, and PaO_2_) compared to the control group at both 4 weeks of treatment and 3-month follow-up. This therapeutic effect may be attributed to anticoagulation-induced improvements in pulmonary microcirculation perfusion and reduced alveolar-capillary barrier damage. Previous studies have confirmed that anti-MDA5 antibodies activate vascular endothelial cells and promote microthrombosis (Huaman Wu and Deng, 2024); anticoagulation therapy may mitigate disease progression by interrupting this pathological process. Concurrently, the treatment group showed marked reductions in inflammatory markers (CRP, LDH, and CK), suggesting that anticoagulation exerts synergistic anti-inflammatory effects by modulating the “coagulation-inflammation” cascade (as supported by Yuetong Xu (2023)). Regarding safety, the incidences of liver/kidney dysfunction and fungal infections were significantly lower in the combined anticoagulation group, indicating potential multi-organ protective effects. Most notably, anticoagulation therapy substantially reduced acute exacerbation incidence. Logistic regression analysis further identified non-anticoagulation treatment, decreased PaO_2_, elevated inflammatory markers, and concurrent infections as independent risk factors for acute exacerbation (Yao et al., 2025). These findings align closely with Hattori et al.’s conclusions regarding RP-ILD prognostic factors (Hattori et al., 2025).

This study observed that combined anticoagulant therapy significantly improved the prognosis of patients, and the mechanism may stem from the synergistic effects of multiple pathways.

Firstly, improving pulmonary microcirculation is crucial. The characteristic alveolar capillary microthrombosis in anti-MDA5 antibody-positive interstitial lung disease (ILD) can lead to gas exchange disorders and tissue hypoxia. Anticoagulant drugs effectively restore the patency and perfusion of pulmonary microvessels by inhibiting thrombus formation, which reasonably explains the significant improvement in PaO_2_ and DLCO%.

Secondly, regulating the “coagulation–inflammation” cascade is essential. Activated coagulation factors (such as factor Xa) are themselves potent pro-inflammatory mediators. Drugs like rivaroxaban can directly inhibit the pro-inflammatory activity of such factors while anticoagulating, thus breaking the “coagulation-inflammation” vicious cycle. The subgroup analysis of non-infected patients in this study confirmed its independent anti-inflammatory effect.

Finally, there is potential multi-organ protection. Microthrombosis is a systemic process. Anticoagulant therapy may indirectly protect liver and kidney functions by alleviating systemic microvascular lesions, which is consistent with the reduced incidence of liver and kidney function abnormalities observed in our study.

Furthermore, our subgroup analysis of patients without infections confirmed that the observed reduction in inflammatory markers was attributable to the anticoagulation therapy itself, thereby strengthening the causal inference.

The natural history of anti-MDA5 antibody-positive ILD is characterized by a rapid and often fatal progression, defining a critical therapeutic window within the first few months after diagnosis. The primary objective of intervention during this period is to urgently halt the inflammatory cascade and prevent irreversible fibrotic lung damage. Therefore, the assessment of a treatment’s ability to improve key physiological and inflammatory parameters within this early, high-risk phase is of paramount clinical importance. Our study, with its focus on 3-month outcomes, was specifically designed to evaluate the efficacy of combined anticoagulation in achieving this urgent early goal.

While this study provides valuable clinical insights into combined anticoagulation therapy for anti-MDA5 antibody-positive ILD, several limitations should be acknowledged. As a single-center retrospective analysis with a moderate sample size (n = 100) and relatively short follow-up period (3 months), the findings may be influenced by selection bias and limited statistical power for subgroup analyses. Furthermore, the non-randomized allocation of anticoagulation therapy introduces potential confounding factors, and the study design did not permit direct comparison between different anticoagulants (enoxaparin vs. rivaroxaban). This study is limited by its retrospective design, which precluded a systematic comparison of key prognostic biomarkers such as ferritin and anti-MDA5 antibody titers between groups at baseline. Although the groups were well-matched in demographics, disease duration, and baseline pulmonary function, future prospective studies should ensure the collection and reporting of these critical variables to further validate our findings. Besides, due to the retrospective nature, we could not standardize or minutely track the tapering schedule of glucocorticoids.

However, our supplementary analysis showed comparable cumulative steroid doses and doses at key timepoints between groups, we cannot fully rule out the potential confounding effect of differential steroid tapering speed on outcomes such as infection rates. Future prospective studies should meticulously record steroid dosing to account for this potential confounder. These methodological constraints highlight the need for future multicenter randomized controlled trials with larger cohorts, longer follow-up durations, and head-to-head comparisons of anticoagulation regimens to validate our findings and optimize treatment protocols. Furthermore, although we optimized the multivariate logistic regression model by strictly limiting the number of variables, the finite number of acute exacerbation events meant the Events Per Variable (EPV) ratio remained suboptimal, which may affect the precision of the model estimates. Future studies with larger sample sizes are needed to further validate these risk factors.

Finally, this study did not systematically analyze serial changes in coagulation biomarkers (such as D-dimer, PT, and APTT) in response to therapy. While the observed clinical improvements strongly support the biological efficacy of combined anticoagulation, future mechanistic studies are warranted to quantitatively document the modulation of the coagulation pathway and its direct correlation with inflammatory abatement and clinical outcomes.

## Conclusion

In conclusion, in the treatment of anti-MDA5 antibody-positive associated interstitial lung disease, combined anticoagulation therapy can not only significantly improve pulmonary function and suppress the inflammatory response but also effectively reduce the incidence of complications and the risk of acute exacerbation, demonstrating important clinical application prospects. This comprehensive treatment strategy offers new evidence-based medical support for improving the prognosis of such patients.

## Data Availability

The original contributions presented in the study are included in the article/Supplementary Material, further inquiries can be directed to the corresponding authors.
