# Supplementary material for: Clinical application of combined anticoagulation therapy in anti-MDA5 antibody-positive associated interstitial lung disease-a retrospective study
Source: Front Pharmacol. 2025 Dec 1;16:1662306. doi: 10.3389/fphar.2025.1662306 (PMC12702877; doi:10.3389/fphar.2025.1662306)
Supplement: Supplementary file 1 [file Supplementaryfile1.docx]

**Supporting information**

**
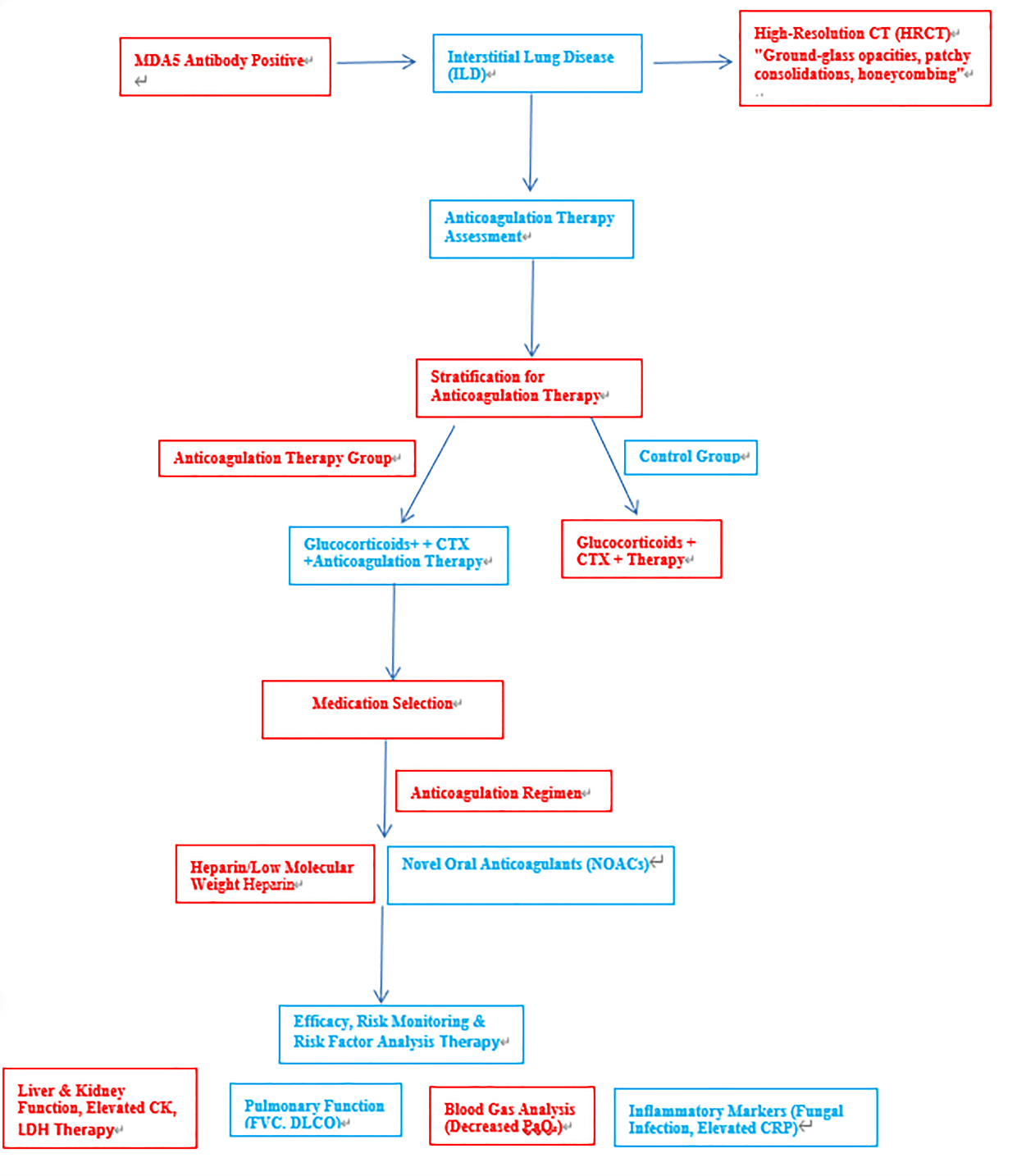
**

**Figure S1**. Study flowchart outlining patient selection, treatment stratification, and outcome assessment for anti-MDA5 antibody-positive interstitial lung disease (ILD) patients receiving combined anticoagulation therapy.

**Table S1.** 2011 ATS/ERS/JRS/ALAT Diagnostic Criteria for IPF

| **Feature** | **Manifestation** |
| --- | --- |
| Clinical Presentation and Signs | Unexplained exertional dry cough, chest tightness, or shortness of breath; auscultation reveals Velcro rales at the lung bases. |
| Imaging Findings | Chest High-Resolution CT (HRCT) shows manifestations of pulmonary interstitial fibrosis, such as ground-glass opacities, patchy shadows, or honeycombing. |
| Pulmonary Function Tests | Restrictive ventilatory dysfunction and/or impaired diffusing capacity. |
| Lung Biopsy | Lung tissue biopsy findings consistent with the pathological pattern of Usual Interstitial Pneumonia (UIP). |
| Exclusion of Other Conditions | Exclusion of tuberculosis, emphysema, and other known causes of ILD. |

Diagnostic Requirement: Fulfillment of at least 2 out of the first 4 criteria, with the imaging findings being a mandatory requirement.
